# Clinical significance of radiological usual interstitial pneumonia pattern in primary Sjögren syndrome-associated interstitial lung disease

**DOI:** 10.3389/fimmu.2026.1811649

**Published:** 2026-06-26

**Authors:** Guanning Zhong, Naihui Wan, Shuyuan Wang, Weiwei Xie, Congcong Zhang, Xiaorui Ding, Yan Li, Yingwei Zhang, Jinghong Dai

**Affiliations:** 1 Department of Pulmonary and Critical Care Medicine, Nanjing Drum Tower Hospital, Affiliated Hospital of Medical School, Nanjing University, Nanjing, China; 2 Department of General Practice, The Second People’s Hospital of Lianyungang, Lianyungang, China; 3 Department of Pulmonary and Critical Care Medicine, Renji Hospital, Shanghai Jiao Tong University School of Medicine, Shanghai, China

**Keywords:** idiopathic pulmonary fibrosis (IPF), interstitial lung disease (ILD), primary Sjögren syndrome (pSS), prognosis, usual interstitial pneumonia (UIP)

## Abstract

**Background:**

The radiological usual interstitial pneumonia (UIP) pattern demonstrates heterogeneity across different underlying etiologies. This study aimed to characterize its clinical and prognostic impact in primary Sjögren’s syndrome-associated interstitial lung disease (pSS-ILD) and compare these features with those in idiopathic pulmonary fibrosis (IPF).

**Methods:**

Consecutive patients with pSS-ILD and IPF were enrolled. Clinical characteristics were compared between pSS-ILD patients with (UIP-pSS) and without (non-UIP-pSS) radiological UIP, as well as between UIP-pSS and IPF patients. Risk factors for UIP pattern were identified by logistic regression. Survival was compared using log-rank test.

**Results:**

In total, 558 pSS-ILD patients and 199 IPF patients were included. Radiological UIP pattern was identified in 18.8% of the pSS-ILD cohort. Compared to non-UIP-pSS patients, UIP-pSS patients were older, comprised more male patients and smokers (all p<0.05), and showed a greater prevalence of pulmonary hypertension (37.1% vs 20.5%, p=0.003). They also exhibited lower lung diffusing capacity for carbon monoxide (DLco) % predicted (p=0.043), increased composite physiologic index (CPI; p=0.016), and higher Gender-Age-Physiology (GAP) index (p=0.002). Also, UIP-pSS patients had increased 1-year (16.2% vs 6.8%, p=0.002), 3-year (34.4% vs 17.7%, p=0.001), and 5-year mortality (55.7% vs 35.0%, p=0.002). When compared to IPF controls, UIP-pSS patients demonstrated higher DLco % predicted (p<0.001), lower CPI (p=0.009) and GAP index (p<0.001), and longer median survival (66.0 vs 40.0 months, p<0.001).

**Conclusions:**

The radiological UIP pattern correlated with impaired pulmonary function and increased mortality in pSS-ILD. However, compared to IPF patients, individuals with UIP-pSS exhibited better pulmonary function and prognosis.

## Introduction

Primary Sjögren’s syndrome (pSS) is a common systemic autoimmune disorder characterized by inflammation of the exocrine glands, predominantly manifesting as sicca symptoms including xerostomia and xerophthalmia (1). pSS frequently presents with systemic involvement (2), including interstitial lung disease (ILD), a common extra-glandular manifestation associated with reduced quality of life, impaired pulmonary function, and increased mortality (3, 4). The high-resolution computed tomography (HRCT) manifestations of pSS-associated ILD (pSS-ILD) are heterogeneous, including non-specific interstitial pneumonia (NSIP), usual interstitial pneumonia (UIP), organizing pneumonia (OP), and lymphocytic interstitial pneumonia (LIP), which vary in prognostic implications (5).

A recent meta-analysis demonstrated a 44% pooled prevalence of radiological UIP in pSS-ILD (6), yet its clinical and prognostic implications remain insufficiently characterized due to limited pSS-specific data in previous CTD-ILD cohorts (5). Evidence suggests that pSS-ILD patients with radiological UIP may have better survival than IPF controls (7), but its disease-specific implications remain to be investigated in large-scale cohorts.

In this study, consecutive patients with pSS-ILD were enrolled from our center, and their demographic characteristics, comorbidities, laboratory parameters, HRCT images, pulmonary function tests, and survival data were collected. Comparisons of clinical characteristics and prognosis were made between pSS-ILD patients with (UIP-pSS) and without (non-UIP-pSS) radiological UIP pattern. A subgroup analysis within the UIP-pSS cohort was performed to compare definite versus probable UIP pattern. Consecutive IPF patients from our center were also included as historical controls for comparative analyses between UIP-pSS and IPF patients.

## Methods

### Patient population

Clinical information of consecutive patients diagnosed with pSS-ILD from November 2007 to November 2022 in the Electronic Medical Record (EMR) system of Nanjing Drum Tower Hospital was systematically reviewed. pSS was diagnosed according to the 2002 revised American-European classification criteria (AECG) or the 2016 American College of Rheumatology/European League Against Rheumatism (ACR/EULAR) classification criteria. pSS-ILD was diagnosed through multidisciplinary discussion (MDD) involving rheumatologists, pulmonologists, radiologists, and pathologists, based on integrated assessment of clinical manifestations, functional tests, HRCT findings, and lung biopsy (if available). Consecutive patients diagnosed with IPF from December 2011 to April 2022 were included as historical controls (IPF group). IPF was diagnosed through MDD involving pulmonologists, radiologists, and pathologists, according to the ATS/ERS/JRS/ALAT guidelines. Patients with other connective tissue diseases (CTDs), missing baseline chest HRCT scans, or with follow-up duration <1 year were excluded.

This study complied with the Declaration of Helsinki and was approved by the Ethics Committee of Nanjing Drum Tower Hospital, Affiliated Hospital of Medical School, Nanjing University (protocol number 2022-067-02, March 28, 2022).

### Chest HRCT assessment

Non−contrast volumetric HRCT scans were acquired during full inspiration with a slice thickness of 1.25 mm. Images were interpreted on a PACS workstation using standard lung window settings (level –600 to –700 HU, width 1500 HU).

All scans were independently reviewed by a pulmonologist and a thoracic radiologist, both blinded to clinical data except for sex and age (due to EMR retrieval). The discordant cases were subsequently reviewed and resolved by consensus.

All scans were categorized into the following patterns: definite UIP, probable UIP, NSIP, OP, LIP, NSIP with OP overlap (NSIP-OP), and unclassified. Definite UIP is characterized by a subpleural, basal-predominant, heterogeneous distribution with honeycombing, with or without traction bronchiectasis or bronchiolectasis. Probable UIP shows a similar distribution with reticular pattern and peripheral traction bronchiectasis or bronchiolectasis, which may include mild ground-glass opacification (GGO) (8). NSIP is defined as basal predominant reticular abnormalities with traction bronchiectasis, peri-bronchovascular extension and subpleural sparing, frequently associated with ground-glass attenuation. OP is defined as bilateral patchy areas of consolidation with a subpleural and lower lung zone predominance. NSIP with OP overlap is defined as basal predominant consolidation, often peri-diaphragmatic, associated with features of fibrosis (e.g. traction bronchiectasis, reticular abnormality or lower lobe volume loss). LIP is defined as predominantly peri-bronchovascular cysts, with or without ground glass opacities or reticular abnormalities (9).

Patients with definite UIP pattern or probable UIP pattern on HRCT were assigned to the UIP-pSS group and others to the non-UIP-pSS group. The UIP-pSS group was then divided into definite UIP-pSS and probable UIP-pSS subgroups.

### Clinical information

Demographic characteristics included sex, age at diagnosis, smoking history, and disease duration from respiratory symptom onset to ILD diagnosis. Comorbidities were recorded, including hypertension, diabetes mellitus, pathologically confirmed malignancies, and pulmonary hypertension (PH). Based on the 2022 ESC/ERS guidelines for PH (10) and a meta-analysis of echocardiographic diagnostic thresholds (11), PH was defined as a systolic pulmonary artery pressure (sPAP) >36 mmHg.

Laboratory parameters at initial hospital admission for ILD were recorded, including white blood cell (WBC) counts, neutrophil counts, lymphocyte counts, hemoglobin (Hb), platelet (PLT) counts, albumin (ALB), globulin (GLOB), albumin-globulin ratio (A/G), C-reactive protein (CRP), lactate dehydrogenase (LDH), erythrocyte sedimentation rate (ESR), carcinoembryonic antigen (CEA), Cytokerantin-19-fragment (CYFRA21-1), and neuronal enolase (NSE). Positive autoantibodies were recorded, including antinuclear antibody (ANA), anti-Sjögren’s syndrome A (anti-SSA) antibody, anti-Sjögren’s syndrome B (anti-SSB) antibody, anti-Ro52 antibody, antiribonucleoprotein (anti-RNP) antibody, and rheumatoid factor (RF). The ANA titer of ≥ 1: 100 was regarded as positive. Isolated anti-Ro52 was defined as positive anti-Ro52 antibodies without concurrent anti-SSA antibodies.

Pulmonary function tests (PFTs) within 6 months of initial hospitalization were collected, including forced vital capacity (FVC) % predicted, forced expiratory volume in one second (FEV_1_) % predicted, and carbon monoxide diffusing capacity (DLco) % predicted. The composite physiologic index (CPI), which integrates ventilatory and diffusing capacity parameters was calculated as follows: CPI = (0.65 * DLco % predicted) – (0.53 * FVC % predicted) + (0.34 * FEV_1_% predicted).

The Gender-Age-Physiology (GAP) index was calculated by integrating patient gender (male:1, female:0), age (≤60 years:0, 61–65 years:1, >65 years:2), FVC % predicted (>75%:0, 50-75%:1, <50%:2), and DLco % predicted (>55%:0, 36-55%:1, ≤35%:2, unable to perform:3).

The EULAR Sjögren’s syndrome disease activity index (ESSDAI) at the initial hospitalization, which includes 12 domains (constitutional, lymphadenopathy and lymphoma, glandular, articular, cutaneous, respiratory, renal, muscular, peripheral nervous system (PNS), central nervous system (CNS), hematological, and biological), was rated for each patient.

Vital status was obtained from medical records or telephone follow-ups. Survival was calculated from the initial hospital admission to all-cause deaths. The follow-ups were ceased until November 2023.

### Statistical analysis

The Cohen’s Kappa statistic was calculated to assess inter-observer agreement for initial UIP pattern classification. Continuous variables were compared using the Mann-Whitney U test and presented as median with interquartile range (IQR). Qualitative data were compared using the Chi-square test or Fisher’s exact test and presented as counts with percentages. Survival was plotted using the Kaplan–Meier method and compared with the log-rank test. Multicollinearity was assessed using variance inflation factor (VIF), with a VIF > 5 indicating collinearity. Binary logistic regression was utilized to identify risk factors. Multivariable Cox proportional hazards regression was performed to identify independent predictors of survival. Analyses were performed using SPSS version 25.0 and GraphPad Prism version 9.5. Two-sided P-values less than 0.05 were deemed statistically significant.

## Results

### Clinical characteristics of UIP-pSS versus non-UIP-pSS

**Figure 1:**
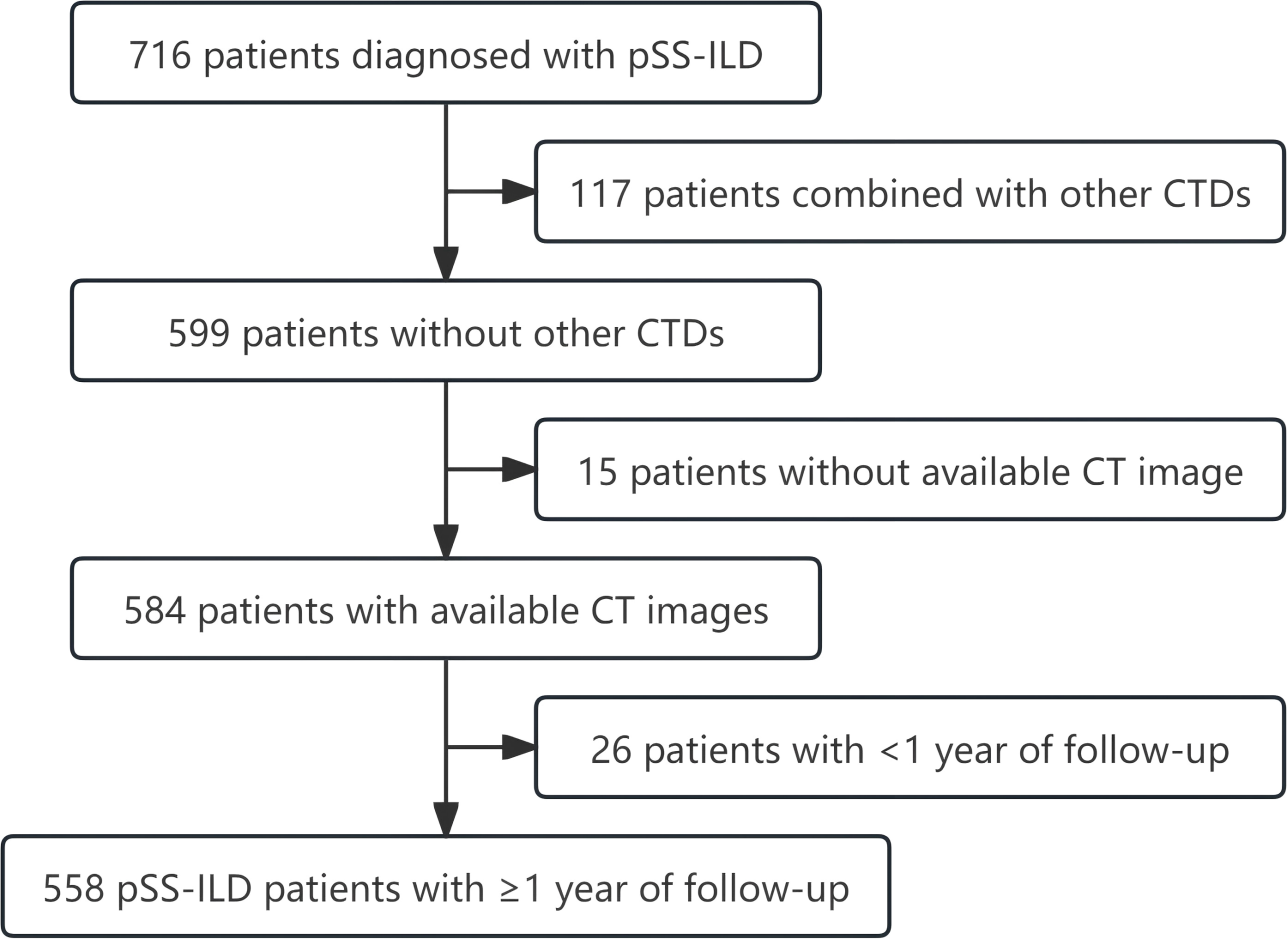
Flow chart of patient selection.

**Figure 2:**
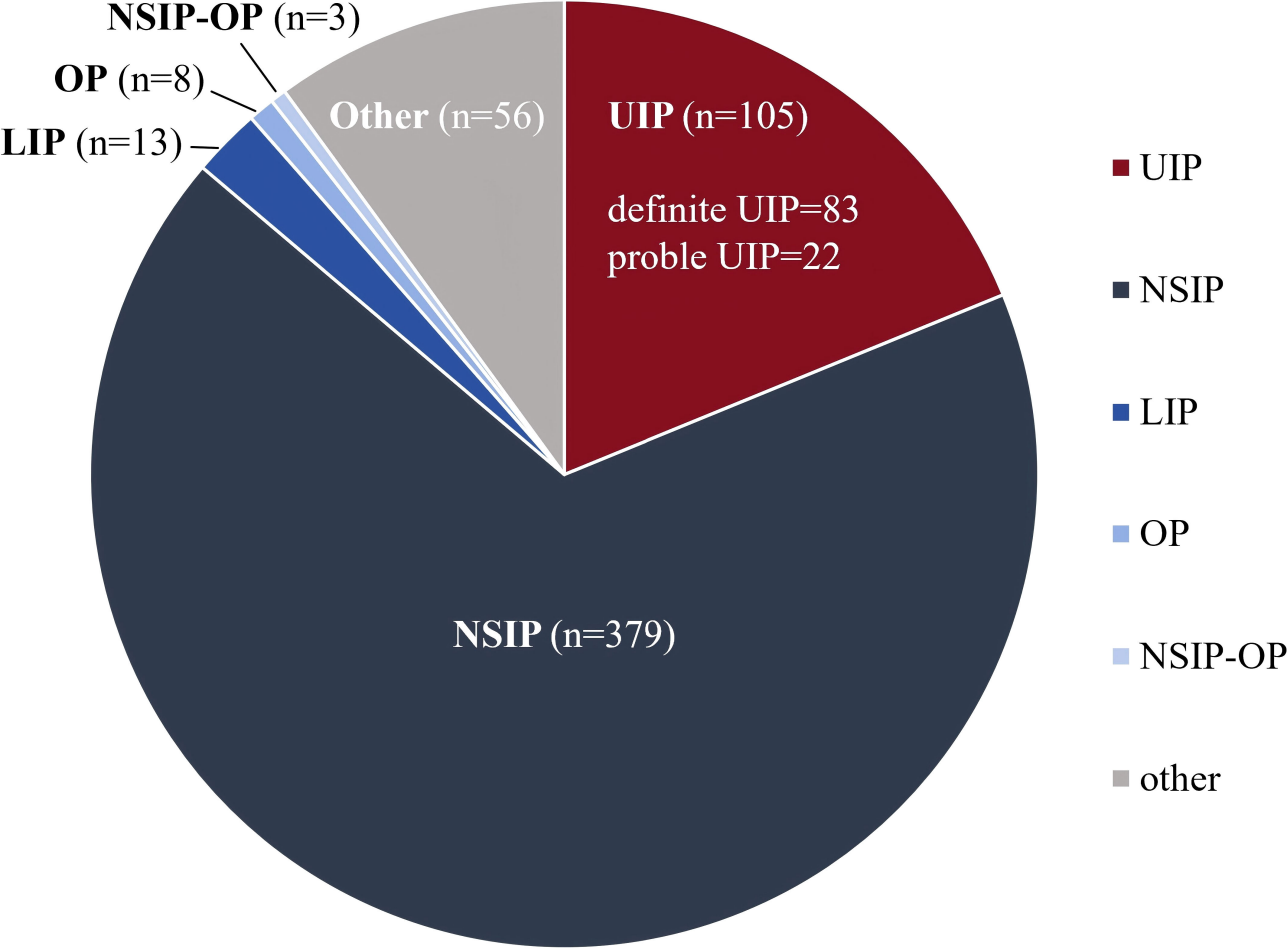
Imaging patterns of the pSS-ILD cohort.

In total, 588 patients with pSS-ILD were enrolled (**Figure 1**). NSIP (n=379, 67.9%) was the most common HRCT pattern, followed by UIP (n=105, 18.8%), LIP (n=13, 2.3%), OP (n=5, 0.9%), and NSIP-OP (n=3, 0.5%). The radiological classification of UIP versus non-UIP showed high inter-observer agreement (Cohen’s kappa=0.954, P < 0.001). Accordingly, 105 patients were classified into the UIP-pSS group and 453 into the non-UIP-pSS group (**Figure 2**). Baseline clinical characteristics are shown in **Table 1**. The UIP-pSS group were older at diagnosis (68.0 vs 61.0, p<0.001) and had more male patients (33.3% vs 19.9%, p=0.003) and smokers (19.0% vs 8.2%, p=0.001) than non-UIP-pSS group. In 407 patients with available echocardiography data (70 in the UIP-pSS group and 337 in the non-UIP-pSS group), PH was more prevalent in the UIP-pSS group than in the non-UIP-pSS group (37.1% vs 20.5%, p=0.003).

**Table 1:** Comparison between the UIP-pSS and non-UIP-pSS groups.

Variables	UIP-pSS	Non-UIP-pSS	P-value
Demographics			
Male	35 (33.3%)	90 (19.9%)	**0.003**
Age (years)	68.0 (59.0, 74.0)	61.0 (52.0, 69.0)	**<0.001**
Smoking history	20 (19.0%)	37 (8.2%)	**0.001**
Disease duration	12.0 (2.0-36.0)	4.0 (1.0-24.0)	**<0.001**
Comorbidities			
Hypertension	41 (39.0%)	146 (32.2%)	0.182
Diabetes Mellitus	18 (17.1%)	85 (18.8%)	0.700
Cancer	13 (12.4%)	34 (7.5%)	0.105
PH	26 (37.1%)	69 (20.5%)	**0.003**
Laboratory examinations			
WBC (*10^9^/L)	6.3 (4.6-8.7)	6.1 (4.7-8.4)	0.823
neutrophil (*10^9^/L)	3.8 (2.6-5.7)	3.8 (2.7-6.0)	0.537
lymphocyte (*10^9^/L)	1.6 (1.2-2.2)	1.4 (1.1-1.9)	**0.004**
Hb (g/L)	123.0 (113.5-135.0)	125.0 (115.0-137.0)	0.388
Plt (*10^9^/L)	196.0 (153.0-244.0)	197.0 (151.5-252.5)	0.920
ALB (g/L)	36.7 (34.7-38.9)	37.4 (34.7-40.0)	0.057
GLOB (g/L)	29.4 (26.4-34.9)	28.3 (25.1-32.7)	**0.042**
A/G	1.25 (1.03-1.39)	1.32 (1.11-1.53)	**0.014**
CRP (mg/L)	4.8 (3.0, 19.0)	4.4 (2.7, 9.7)	0.262
LDH (U/L)	239.0 (194.5-278.0)	224.0 (190.0-286.5)	0.611
ESR (mm/h)	28.0 (16.0, 50.0)	24.0 (13.0, 43.0)	0.092
CEA (ng/ml)	1.8 (1.1, 3.2)	1.7 (0.8, 2.8)	0.150
CYFRA21-1 (ng/ml)	3.9 (3.0, 6.0)	3.4 (2.6, 5.5)	**0.022**
NSE (ng/ml)	14.3 (10.9, 17.8)	14.9 (12.5, 18.5)	0.194
ANA	87 (75.2%)	357 (82.1%)	0.111
Anti-SSA	29 (27.6%)	161 (35.5%)	0.123
Anti-SSB	15 (14.3%)	62 (13.7%)	0.873
Anti-Ro52	48 (45.7%)	267 (58.9%)	**0.014**
Isolated anti-Ro52	26 (24.8%)	146 (32.2%)	0.135
RF	9 (8.6%)	55 (12.1%)	0.301
ESSDAI			
Total ESSDAI	15.0 (12.0-19.0)	15.0 (11.0-19.0)	0.133
ESSDAI without pulmonary domain	4.0 (2.0-7.0)	4.0 (2.0-8.0)	0.432
Pulmonary function tests			
FVC % predicted	65.9 (54.8, 79.8)	67.5 (53.9, 83.3)	0.523
FEV_1_% predicted	73.5 (61.9, 88.3)	73.7 (58.8, 89.3)	0.958
DLco % predicted	47.4 (39.0, 60.8)	54.4 (40.5, 67.0)	**0.043**
CPI	49.7 (43.3, 58.6)	44.5 (33.5, 55.2)	**0.016**
GAP index	3.0 (2.0, 4.0)	2.0 (1.0, 3.0)	**0.002**
Outcomes			
1-year mortality	17 (16.2%)	31 (6.8%)	**0.002**
3-year mortality	31 (34.4%)	59 (17.7%)	**0.001**
5-year mortality	39 (55.7%)	79 (35.0%)	**0.002**
Median survival	66.0	115.0	**<0.001**

PH: pulmonary hypertension, analyzed in patients with available echocardiography data, n=407 (70 UIP-pSS, 337 non-UIP-pSS); ESR: n=527 (99 UIP-pSS, 428 non-UIP-pSS); CEA: n=448 (93 UIP-pSS, 355 non-UIP-pSS); CYFRA21-1: n=440 (92 UIP-pSS, 348 non-UIP-pSS); NSE: n=438 (91 UIP-pSS, 347 non-UIP-pSS); FVC % predicted: n=260 (59 UIP-pSS, 201 non-UIP-pSS); FEV_1_% predicted: n=248 (56 UIP-pSS, 193 non-UIP-pSS); DLco % predicted: n=215 (49 UIP-pSS, 166 non-UIP-pSS); CPI: n=205 (44 UIP-pSS, 161 non-UIP-pSS); GAP index: n=214 (47 UIP-pSS, 167 non-UIP-pSS). Bold values indicate P < 0.05.

In terms of laboratory parameters, the UIP-pSS group had higher levels of lymphocyte counts (1.6 vs 1.4, p=0.004), GLOB (29.4 vs 28.3, p=0.004), and CYFRA21-1 (3.9 vs 3.4, p=0.022), while non-UIP-pSS group had higher level of A/G (1.32 vs 1.25, p=0.014) and higher anti-Ro52 antibody positivity (58.9% vs 45.7%, p=0.014). Besides, the UIP-pSS group had significantly lower DLco % predicted (47.4 vs 54.4, p=0.043) and higher CPI (49.7 vs 44.5, p=0.016). They also exhibited a higher GAP index than the non-UIP-pSS group (3.0 vs 2.0, p=0.002). No significant difference was observed in baseline ESSDAI between the two groups (p>0.050).

**Figure 3:**
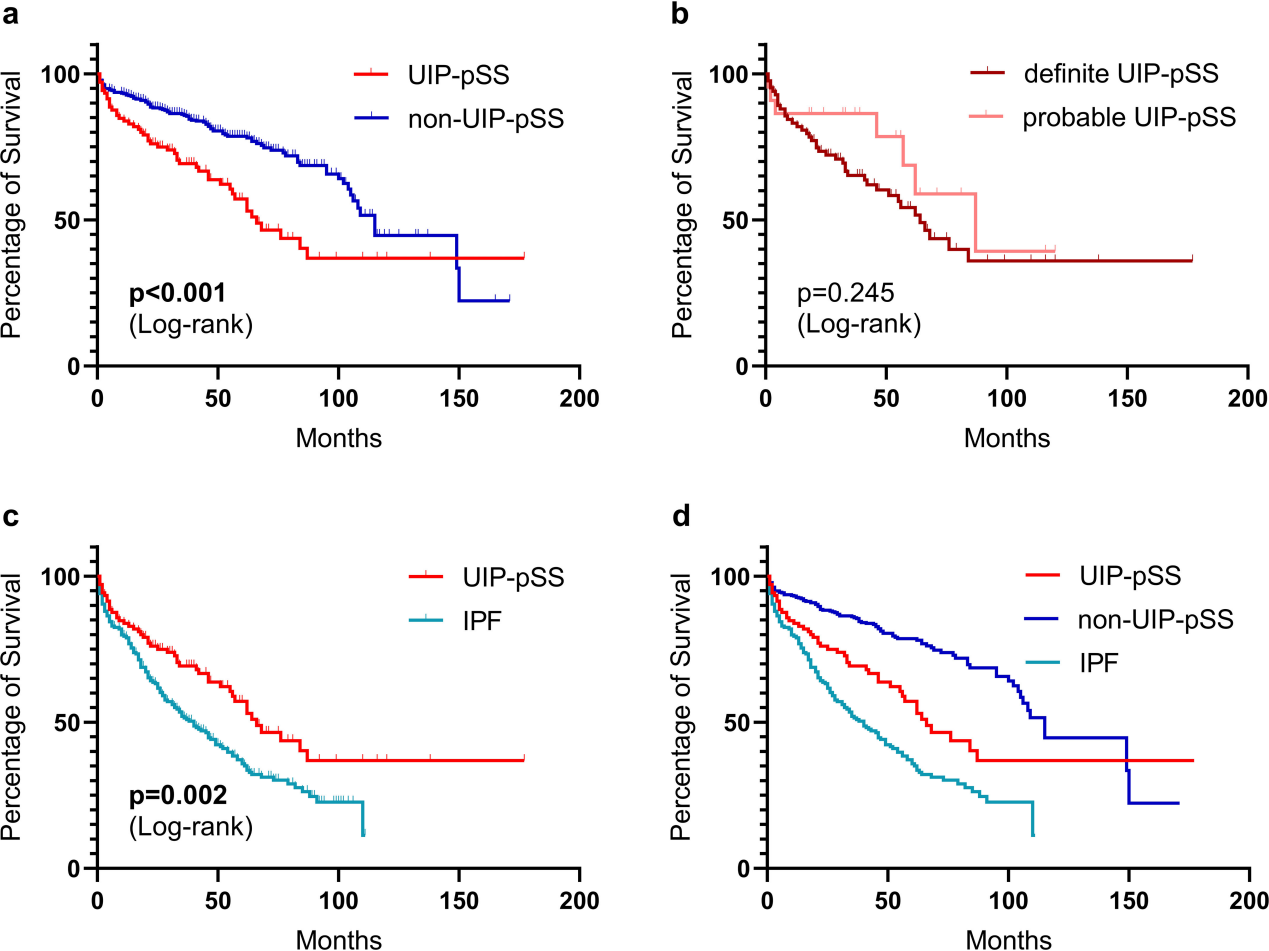
K-M curves of the pSS-ILD patients and IPF patients. **(A)** K-M curves of UIP-pSS group and non-UIP-pSS group; **(B)** K-M curves of definite UIP-pSS group and probable UIP-pSS group; **(C)** K-M curves of UIP-pSS group and IPF group. **(D)** K-M curves of all three groups (UIP-pSS, non-UIP-pSS, and IPF).

A total of 423 patients had available 3-year survival data (90 in the UIP-pSS group and 333 in the non-UIP-pSS group), and 296 patients had available 5-year survival data (70 in the UIP-pSS group and 226 in the non-UIP-pSS group). Compared with the non-UIP-pSS group, the UIP-pSS group had significantly higher 1-year mortality (16.2% vs 6.8%, p=0.002), 3-year mortality (34.4% vs 17.7%, p=0.001), and 5-year mortality (55.7% vs 35.0%, p=0.002). The median survival of the UIP-pSS group was significantly shorter than that of the non-UIP group (66.0 vs 115.0 months, p<0.001) (**Figure 3**).

### Clinical characteristics of definite versus probable UIP-pSS

Among UIP-pSS cases, definite UIP pattern was observed in 83 patients (79.0%) while probable UIP pattern was observed in 22 patients (21.0%) (**Figure 1**). As shown in **Table 2**, no significant difference was found in demographic characteristics, baseline PFTs, and prognosis between the definite UIP-pSS group and probable UIP-pSS group (all p>0.050).

**Table 2:** Comparison between the definite UIP-pSS and probable UIP-pSS groups.

Variables	Definite UIP-pSS	Probable UIP-pSS	P-value
Demographics			
Men	27 (32.5%)	8 (36.4%)	0.735
Age (years)	69.0 (60.0, 74.0)	62.5 (58.0, 71.5)	0.252
Smoking history	18 (21.7%)	2 (9.1%)	0.233
Disease duration	12.0 (2.0-36.0)	12.0 (1.0-30.0)	0.561
Pulmonary function tests			
FVC % predicted	66.7 (55.8-78.9)	65 (45.6, 83.9)	0.978
FEV_1_% predicted	73.8 (63.3, 89.4)	69.0 (48.6, 83.6)	0.298
DLco % predicted	47.5 (41.3, 61.0)	42.5 (34.6, 60.0)	0.497
CPI	48.6 (43.2, 57.3)	52.1 (40.2, 63.0)	0.456
GAP index	3.0 (2.0, 4.0)	2.5 (1.0, 3.0)	0.123
Outcomes			
1-year mortality	14 (16.9%)	3 (13.6%)	0.999
3-year mortality	28 (37.8%)	3 (18.8%)	0.145
5-year mortality	34 (58.6%)	5 (41.7%)	0.282
Median survival	64.0	87.0	0.245

FVC % predicted: n=60 (48 definite UIP-pSS, 12 probable UIP-pSS); FEV_1_% predicted: n=56 (45 definite UIP-pSS, 11 probable UIP-pSS); DLco % predicted: n=47 (37 definite UIP-pSS, 10 probable UIP-pSS); CPI: n=44 (35 definite UIP-pSS, 9 probable UIP-pSS); GPA index: n=47 (37 definite UIP-pSS, 10 probable UIP-pSS).

### Risk factors for radiological UIP in pSS-ILD

Binary logistic regression was utilized to identify potential risk factors for radiological UIP in pSS-ILD (**Table 3**). Univariate regression showed that male (OR = 2.017, 95%CI 1.264-3.216), age (OR = 1.042, 95%CI 1.022-1.061), smoking history (OR = 2.645, 95%CI 1.464-4.781), disease duration (OR = 1.005, 95%CI 1.000-1.010), lymphocyte counts (OR = 1.430, 95%CI 1.083-1.887), A/G (OR = 0.439, 95%CI 0.217-0.591), and anti-Ro52 antibody (OR = 0.587, 95%CI 0.383-0.899) were significant factors.

**Table 3:** Risk factors for radiological UIP in pSS-ILD patients.

Variables	Univariate			Multivariate		
	P-value	OR	95%CI	P-value	OR	95%CI
Male	**0.003**	2.107	1.264-3.216	0.786	1.089	0.589-2.014
Age	**<0.001**	1.042	1.022-1.061	**0.002**	1.031	1.011- 1.052
Smoking history	**0.001**	2.645	1.464-4.781	0.054	2.092	0.988-4.431
Disease duration	**0.024**	1.005	1.001-1.010	0.050	1.005	1.000-1.009
WBC (*10^9^/L)	0.999	1.000	0.939-1.065			
neutrophil (*10^9^/L)	0.331	0.962	0.889-1.040			
lymphocyte (*10^9^/L)	**0.012**	1.430	1.083-1.887	**0.016**	1.418	1.068-1.883
Hb (g/L)	0.747	0.998	0.986-1.010			
Plt (*10^9^/L)	0.880	1.000	0.998-1.003			
ALB (g/L)	0.148	0.962	0.912-1.014			
GLOB (g/L)	0.209	1.018	0.990-1.047			
A/G	**0.023**	0.439	0.217-0.591	**0.028**	0.429	0.201-0.914
CRP (mg/L)	0.415	1.004	0.995-1.013			
LDH (U/L)	0.336	0.999	0.996-1.015			
ESR (mm/h)	0.178	1.006	0.997-1.014			
CEA (ng/ml)	0.678	1.024	0.917-1.143			
CYFRA21-1 (ng/ml)	0.764	1.008	0.958-1.061			
NSE (ng/ml)	0.310	0.980	0.943-1.019			
ANA	0.075	0.632	0.381-1.048			
Anti-SSA	0.124	0.692	0.433-1.106			
Anti-SSB	0.873	1.051	0.572-1.932			
Anti-Ro52	**0.014**	0.587	0.383-0.899	0.153	0.718	0.456-1.131
Isolated anti-Ro52	0.137	0.692	0.426-1.124			
RF	0.304	0.678	0.324-1.421			

ESR: n=527 (99 UIP-pSS, 428 non-UIP-pSS); CEA: n=448 (93 UIP-pSS, 355 non-UIP-pSS); CYFRA21-1: n=440 (92 UIP-pSS, 348 non-UIP-pSS); NSE: n=438 (91UIP-pSS, 347 non-UIP-pSS). Bold values indicate P < 0.05.

Multivariable logistic regression incorporated clinically recognized confounders including sex, age, smoking history, and disease duration, as well as variables that were significant in univariate analysis. No collinearity was detected (all VIF < 2). Age (OR = 1.031, 95%CI 1.011-1.052), disease duration (OR = 1.005, 95%CI 1.000-1.009), and lymphocyte counts (OR = 1.418, 95%CI 1.068-1.883) were identified as independent risk factors for radiological UIP, while A/G ratio was protective (OR = 0.429, 95%CI 0.201-0.914).

### Clinical characteristics of UIP-pSS versus IPF

In total, 199 IPF historical controls with follow-up for at least 1 year were enrolled. Comparative clinical characteristics of the UIP-pSS and IPF groups are detailed in **Table 4**. The IPF group had more male patients (p<0.001) and smokers (p<0.001) than the UIP-pSS group. Baseline PFTs indicated that the UIP-pSS group had higher DLco % predicted (47.4 vs 37.2, p<0.001) as well as lower CPI (49.7 vs 55.9, p=0.009) and GAP index (3.0 vs 5.0, p<0.001) than the IPF group.

**Table 4:** Comparison between the UIP-pSS and IPF groups.

Variables	UIP-pSS	IPF	P-value
Demographics			
Male	35 (33.3%)	178 (98.4%)	**<0.001**
Age (years)	68.0 (59.0, 74.0)	67 (62.0, 74.0)	0.551
Smoking history	20 (19.0%)	120 (60.3%)	**<0.001**
Disease duration	12.0 (2.0-36.0)	12.0 (3.0-36.0)	0.955
Pulmonary function tests			
FVC % predicted	65.9 (54.8, 79.8)	67.2 (55.8, 82.6)	0.443
FEV_1_% predicted	73.5 (61.9, 88.3)	76.3 (62.4, 89.4)	0.391
DLco % predicted	47.4 (39.0, 60.8)	37.2 (28.4, 47.6)	**<0.001**
CPI	49.7 (43.3, 58.6)	55.9 (48.3, 62.3)	**0.009**
GAPI	3.0 (2.0, 4.0)	5.0 (3.0, 5.5)	**<0.001**
Outcomes			
1-year mortality	17 (16.2%)	41 (20.6%)	0.352
3-year mortality	31 (34.4%)	95 (48.5%)	**0.027**
5-year mortality	39 (55.7%)	120 (69.0%)	**0.049**
Median survival	66.0	40.0	**0.002**

FVC % predicted: n=238 (59 UIP-pSS, 179 IPF); FEV_1_% predicted: n=231 (56 UIP-pSS, 175 IPF); DLco % predicted: n=215 (49 UIP-pSS, 166 IPF); CPI: n=210 (44 UIP-pSS, 166 IPF); GPAI: n=232 (47 UIP-pSS, 185 IPF). Bold values indicate P < 0.05.

Compared to the IPF group, the UIP-pSS group had significantly lower 3-year mortality (34.4% vs 48.5%, p=0.027), lower 5-year mortality (55.7% vs 69.0%, p=0.049), and longer median survival (66.0 vs 40.0 months, p=0.002, **Figure 2**).

A multivariable Cox proportional hazards regression analysis was performed including patients with UIP-pSS and IPF. After adjusting for sex, age, smoking history, and disease duration, IPF diagnosis (HR = 1.762, 95% CI: 1.182–2.628, p=0.005) remained an independent risk factor for poor survival (**Table 5**).

**Table 5:** Multivariable Cox regression analysis of prognostic factors in patients with pSS-UIP and IPF.

Variables	P-value	OR	95%CI
Male	0.336	1.247	0.795-1.955
Age	**0.001**	1.029	1.011-1.047
Smoking history	**0.005**	1.623	1.155-2.280
Disease duration	0.135	1.003	0.999-1.006
IPF	**0.005**	1.762	1.182-2.628

Bold values indicate P < 0.05.

## Discussion

This study revealed that in pSS-ILD, patients with radiological UIP pattern on HRCT were older, more likely to be male patients and smokers, and had a higher prevalence of pulmonary hypertension, more severe pulmonary function impairment, and increased mortality than those without. Within the UIP-pSS cohort, patients with definite versus probable UIP pattern showed similar clinical characteristics and prognosis. Advanced age, prolonged disease duration, elevated lymphocyte count, and reduced albumin-to-globulin ratio were independent risk factors for radiological UIP. Notably, UIP-pSS patients had less severe pulmonary function impairment and lower mortality risk than IPF patients.

Patients exhibiting radiological UIP pattern in pSS-ILD demonstrated more severe impairment of pulmonary diffusion capacity and shorter median survival, highlighting its potential as an adverse prognostic indicator. These findings are consistent with previous studies in other CTD-ILD. In rheumatoid arthritis-associated ILD (RA-ILD), patients with radiological UIP pattern exhibited a 66%-88% elevated mortality risk and accelerated pulmonary function decline than other patterns (12, 13). Similarly, radiological UIP pattern in systemic sclerosis-associated ILD (SSc-ILD) is associated with more rapid disease progression and worse survival (14). In idiopathic inflammatory myopathy-associated ILD (IIM-ILD), the UIP pattern portends poor outcomes, evidenced by 5-year survival rates as low as 33% (15). In a prospective cohort including seven types of CTD-ILD, those exhibiting radiological UIP demonstrated significantly accelerated FVC decline and higher all-cause mortality compared with those with NSIP and OP (5). Notably, despite similar radiological features, IPF patients had significantly worse survival than UIP-pSS patients, consistent with prior evidence that IPF carries a worse prognosis than radiological UIP in CTDs including systemic sclerosis (SSc) (16), RA (17, 18), and pSS (7).

In IPF, patients with probable UIP pattern and definite UIP pattern on HRCT have similar disease behavior and clinical courses (19). In our pSS-ILD cohort, definite UIP pattern also carried similar prognostic significance to probable UIP pattern, indicating that both patterns may reflect a progressive fibroproliferative phenotype. This may underscore the critical importance of early diagnosis and timely antifibrotic therapy in pSS-ILD patients exhibiting UIP features. Robust clinical evidence has demonstrated treatment benefits of antifibrotic therapy across a spectrum of CTD-ILDs, especially in those with progressive fibrosis (20, 21). However, high-level evidence supporting antifibrotic therapy in pSS-ILD remains lacking. Previous studies on pSS-ILD treatment focused mainly on immunomodulatory agents, such as mycophenolate mofetil, azathioprine, and rituximab, which have been shown to improve or stabilize pulmonary function (22). To date, only a few studies have explored antifibrotics in pSS-ILD. A CTD-ILD cohort including 13 pSS-ILD patients (8 with UIP pattern) suggested that the combination of nintedanib and immunosuppressants may provide clinical benefits (23). Another clinical observational study involving 120 pSS-ILD patients reported that pirfenidone provided additional improvements in pulmonary function, yet only patients with NSIP pattern were enrolled (24). The benefit of pirfenidone in UIP-pSS was described in a case report with only 2 patients (25). Collectively, these findings indicate that antifibrotic therapy may have a role in pSS-ILD patients. However, dedicated prospective studies with larger sample sizes and well-defined radiological phenotypes are urgently needed to clarify their efficacy.

To explore factors associated with radiological UIP in pSS-ILD, we performed multivariate logistic regression and identified advanced age as an independent risk factor, suggesting a potential link between UIP and senescence, a key driver of pulmonary fibrosis. Aging-related factors such as telomere shortening and mitochondrial dysfunction can promote the release of senescence-associated secretory phenotype (SASP) and accelerate fibrosis (26, 27). In IPF, senescence markers are upregulated in alveolar epithelial cells (28). Higher lymphocyte count was also identified as an independent UIP risk factor in our cohort. A Large pSS cohort suggested that low lymphocyte count correlates with higher disease activity (29). Mechanistically, lower lymphocyte counts may reflect active autoimmune inflammation and thus correspond to imaging features such as ground-glass opacities, consolidations, and cysts, which are categorized as “alternative diagnosis” rather than UIP according to the ATS/ERS/JRS/ALAT guideline.

The pulmonary domain is a core component of ESSDAI, and previous study showed that higher ESSDAI was associated with the development of ILD (30). However, we did not observe a significant difference in ESSDAI scores between the UIP-pSS and non-UIP-pSS groups. Of note, two retrospective cohort studies have observed a higher prevalence of UIP in non−sicca onset pSS−ILD patients, often in the absence of typical autoantibodies (31, 32). These findings suggest that the pathogenesis of UIP in CTD-ILD may be partially independent of systemic immune activity. Evidence in RA-ILD further supports this hypothesis. In RA-ILD, UIP predominated in ILD-onset patients (33), and shared the *MUC5B* rs35705950 risk allele with IPF (34). A bidirectional Mendelian randomization study further revealed that pulmonary fibrosis may increase the risk of RA, suggesting a reverse causality (35). In contrast, similar mechanistic evidence is currently lacking in pSS-ILD, warranting further investigation.

Large-scale studies indicate that ILD and pulmonary hypertension are common causes of mortality in patients with pSS (36). Although right heart catheterization (RHC) is the gold standard for the diagnosis and hemodynamic stratification of PH, echocardiography has emerged as a useful non−invasive tool for assessing PH risk (10). In our cohort, echocardiography-suspected PH was more frequently identified in patients with the radiological UIP pattern. This finding aligns with prior reports of UIP in SSc-ILD (37). The heightened susceptibility to PH in radiological UIP patients may be attributed to severe diffusion impairment leading to chronic hypoxemia, as well as pulmonary capillary bed destruction and vascular fibrosis secondary to pulmonary fibrosis (38, 39). However, echocardiography cannot help determine the subgroup classification of PH. Therefore, for patients with echocardiography−suspected severe PH, RHC may be necessary and beneficial to guide treatment.

Treatment of PH in CTD-ILD is challenging. The use of pulmonary vasodilators in patients with parenchymal lung disease has been controversial, given concern for worsening hypoxemia due to reversal of hypoxic pulmonary vasoconstriction and pulmonary oedema (40). Current management relies on optimizing ILD and CTD therapy, long−term oxygen, and diuretics, as PH−targeted drugs such as riociguat and ambrisentan are generally avoided in ILD-related PH (10). Timely diagnosis is therefore critical for this patient subset. Given the significant intergroup difference in PH risk, patients with radiologically UIP patterns warrant intensified noninvasive pulmonary arterial pressure monitoring to facilitate early intervention.

Several limitations warrant consideration in our study. First, the single-center, retrospective design inevitably introduces bias and missing data, which may limit the generalizability of the results. Second, the comparisons of baseline clinical characteristics between groups were cross-sectional in nature, making the assessment of longitudinal changes unavailable. Third, patients with a follow-up duration shorter than one year were excluded to reduce censored data and strengthen the reliability of survival analysis, yet this strategy may inevitably introduce potential selection bias. Fourth, the use of historical controls may introduce temporal bias due to partially non-overlapping enrollment periods between the two cohorts.

In conclusion, the radiological UIP pattern in pSS-ILD was characterized by older age, male predominance, higher rates of smoking and pulmonary hypertension, worse pulmonary function, and increased mortality. Notably, UIP-pSS patients had better pulmonary diffusing capacity and longer survival compared with IPF patients.

## Data Availability

The original contributions presented in the study are included in the article/**Supplementary Material**. Further inquiries can be directed to the corresponding authors.

## Supplementary material

Supplementary Table S1 Tolerance and variance inflation factor (VIF) for multicollinearity assessment.
