# Supplementary material for: Clinical significance of radiological usual interstitial pneumonia pattern in primary Sjögren syndrome-associated interstitial lung disease
Source: Front Immunol. 2026 Jun 26;17:1811649. doi: 10.3389/fimmu.2026.1811649 (PMC13350474; doi:10.3389/fimmu.2026.1811649)
Supplement: Supplementary Table S1 — Tolerance and variance inflation factor (VIF) for multicollinearity assessment. [file Table1.docx]

**Table S1** Tolerance and variance inflation factor (VIF) for multicollinearity assessment

| Variable | Tolerance | VIF |
| --- | --- | --- |
| Male | 0.683 | 1.465 |
| Age | 0.913 | 1.095 |
| Smoking history | 0.705 | 1.419 |
| Disease duration | 0.986 | 1.014 |
| Lymphocyte | 0.976 | 1.025 |
| A/G | 0.966 | 1.035 |
| Anti-Ro52 | 0.941 | 1.062 |
